# Increased Risk of Coronary Heart Disease in Patients with Primary Fibromyalgia and Those with Concomitant Comorbidity—A Taiwanese Population-Based Cohort Study

**DOI:** 10.1371/journal.pone.0137137

**Published:** 2015-09-14

**Authors:** Chia-Hsien Su, Jiunn-Horng Chen, Joung-Liang Lan, Yu-Chiao Wang, Chun-Hung Tseng, Chung-Yi Hsu, Lichi Huang

**Affiliations:** 1 Department of Public Health, China Medical University, Taichung, Taiwan; 2 Department of Nursing, China Medical University Hospital, Taichung, Taiwan; 3 School of Medicine, China Medical University, Taichung, Taiwan; 4 Division of Immunology and Rheumatology, Department of Internal Medicine, China Medical University Hospital, Taichung, Taiwan; 5 Management Office for Health Data, China Medical University Hospital, Taichung, Taiwan; 6 Department of Neurology, China Medical University Hospital, Taichung, Taiwan; 7 Graduate Institute of Clinical Medical Science, China Medical University, Taichung, Taiwan; 8 School of Nursing, China Medical University, Taichung, Taiwan; Yokohama City University, JAPAN

## Abstract

**Objectives:**

Fibromyalgia has seldom been associated with coronary heart disease (CHD). The aim of this study was to evaluate the risk of CHD in patients with fibromyalgia.

**Methods:**

We used a dataset of one million participants, systemically scrambled from the Taiwanese national insurance beneficiaries, to identify 61,612 patients with incident fibromyalgia (ICD-9-CM 729.0–729.1) and 184,834 reference subjects matched by sex, age and index date of diagnosis in a 1:3 ratio from 2000 to 2005, with a mean 8.86 ± 2.68 years of follow-up until 2011. Risk of CHD was analyzed by Cox proportional hazard modeling.

**Results:**

Patients with fibromyalgia had a mean age of 44.1 ± 16.5 years. CHD events developed in fibromyalgia patients (n = 8,280; 15.2 per 10^3^ person-years) and reference subjects (n = 15,162; 9.26 per 10^3^ person-years) with a significant incidence rate ratio of 1.64 (95% confidence interval: 1.61–1.68). The adjusted hazard ratio for CHD in fibromyalgia patients relative to reference subjects was 1.47 (1.43–1.51), after adjusting for age, gender, occupation, monthly income, traditional cardiovascular comorbidities, depression and anxiety. We noted that fibromyalgia and cardiovascular comorbidities had a significant interaction effect on CHD risk (*p* for interaction <0.01), which was markedly enhanced in fibromyalgia patients with concomitant comorbidities relative to patients with primary fibromyalgia and reference subjects (no fibromyalgia, no comorbidity).

**Conclusions:**

Our report shows that fibromyalgia patients have an independent risk for CHD development. Fibromyalgia patients with concomitant comorbidities have markedly increased CHD risk relative to those with primary fibromyalgia.

## Introduction

Fibromyalgia is a syndrome with a broad spectrum of symptoms, including chronic widespread pain, non-restorative sleep, overwhelming fatigue, emotional swings, and cognitive dysfunction, with impaired daily social function and life quality [1]. This syndrome involves pain patterns described as hyperalgesia and allodynia, but evidence is still insufficient to support fibromyalgia as an inflammatory or neuropathic disease [2].

The worldwide prevalence of fibromyalgia, according to classification criteria of the American College of Rheumatology (ACR) [3, 4], is ~2–4% of the population [5, 6], with lower prevalence in Asia [7, 8]. Although its prevalence increases with age, fibromyalgia may occur in children [9, 10]. Women are 3 to 7 times more commonly diagnosed with fibromyalgia than men [10]. On the other hand, the recent population-based studies using the modified 2010 classification criteria of American College of Rheumatology [11] reported a higher prevalence (~5–7%) of fibromyalgia [12, 13]. In general rheumatologic clinics, fibromyalgia is second to osteoarthritis as the most common condition [14].

Fibromyalgia commonly presents with comorbidity such as psychiatric diseases (major depressive disorder, anxiety disorder), headache, irritable bowel syndrome, and interstitial cystitis [15–17]. However, fibromyalgia has seldom been associated with coronary heart disease (CHD). An association between fibromyalgia and CHD with subclinical left ventricular dysfunction has been reported in older, female Korean patients [18]. Similarly, Israeli patients who had received coronary catheterization had significantly greater tenderness and higher scores on the fibromyalgia impact questionnaire [19]. The present nationwide population-based cohort study in Taiwan was undertaken to evaluate if there is an independent risk of CHD in patients with fibromyalgia including those with fibromyalgia only and those with concomitant comorbidities.

## Materials and Methods

### Data source

For this population-based cohort study, our main data source was the National Health Insurance Research Database (NHIRD) in Taiwan (S1 Text). This database contains information from registration and reimbursement claims to the National Health Insurance (NHI), started in 1995 and covering over 99% of Taiwan’s population. The informed consent of each individual was not obtained. Therefore, scrambling identification numbers of patients before NHIRD compilation was to protect patient privacy. We obtained a database containing medical reimbursement claims from 1996 to 2011 for 1 million people randomly selected from the NHIRD to represent the whole population. This database was released by the NHI as the Longitudinal Health Insurance Database (LHID) [20]. The age and sex of patients in the LHID and full NHIRD did not differ significantly, and the accuracy and validity of diagnoses in the NHIRD have been confirmed [20, 21]. The ethical review board of the China Medical University in Taiwan (CMU-REC-101-012) approved this study (S2 Text).

### Criteria for selecting subjects

Diseases of interest were coded using the International Classification of Diseases, 9th Revision, Clinical Modification (ICD-9-CM). Disease coding is strictly regulated by the NHI to prevent medical fraud by overbilling for health care or inappropriate charges based on unconfirmed diagnoses. Thus, the NHI regularly monitors coding and claims submitted for reimbursement by all NHI beneficiaries. These precautions for coding errors, misdiagnosis or inappropriate treatment support the reliability of using NHIRD information to explore the risk of CHD in fibromyalgia patients. Among patients in the LHID, 61,711 incident fibromyalgia (ICD-9-CM 729.0–729.1) cases were identified during the study period (2000–2003).

Each patient’s fibromyalgia diagnosis date was defined as its entry date in the LHID. All subjects were followed-up from the entry date until censored, withdrawal from the database, or 31 December 2011. Patients were excluded from the study (n = 99), if they had missing data on age or gender and were diagnosed with CHD before fibromyalgia diagnosis. After these 99 patients were excluded, 61,612 patients with fibromyalgia remained and were ascribed to the case cohort. These patients were matched at baseline by age, gender, and diagnosis date of fibromyalgia in a 1:3 ratio with a reference cohort of non-fibromyalgia subjects in the LHID (n = 184,834; only two non-fibromyalgia reference subject did not qualify for matching criteria).

### Outcome and relevant variables

The primary outcome was the event of CHD with acute coronary syndrome, including clinical conditions of angina pectoris, ischemic heart disease, and coronary atherosclerosis (ICD-9-CM 411.1, 413, 414.0, 414.8, 414.9) during the study period (2000–2011). Relevant variables were age, gender and comorbidities, including type II diabetes mellitus (ICD-9-CM 250), hypertension (ICD-9-CM 401–405), hyperlipidemia (ICD-9-CM 272), congestive heart failure with or without renal disease (ICD-9-CM 402.01, 402.11, 402.91, 404.01, 404.03, 404.11, 404.13, 404.91, 404.93, 428.0), cerebral vascular diseases (ICD-9-CM 430–438), depression (ICD-9-CM 296.2–296.3, 300.4, 311), and anxiety (ICD-9-CM 300.0, 300.2, 300.3, 308.3, 308.91). All comorbidities were defined before the index date of diagnosis.

### Statistical analysis

Differences in demographic characteristics and comorbidities between the case cohort of fibromyalgia patients and the reference cohort were tested by chi-square test for categorical variables or Student’s *t*-test for continuous variables. The gender-, age- and comorbidity-specific incidence rates (per 10^3^ person-years) of CHD between the case and reference cohorts were compared. Person-years were calculated from the entry dates to the censored dates of CHD occurrence, withdrawal from follow-up, or the end of 2011. Incidence rate ratios (IRRs) and 95% confidence intervals (95% CIs) of CHD in fibromyalgia patients relative to reference subjects were estimated using the Poisson regression model. Multivariate adjusted hazard ratios (HRs) and 95% CIs of CHD in fibromyalgia patients relative to the reference subjects were derived by Cox proportional hazard models after adjusting for age, gender, occupation status, monthly income level, and comorbidities. We further compared the risk of CHD development in patients with fibromyalgia by stratification with respect to (1) gender, (2) age, and (3) comorbidities. Age subgroups were stratified into < 35 years, 35–65 years and > 65 years. Differences in the CHD incidence of cohorts, plotted as Kaplan-Meier survival curves, were tested by log-rank test. A *p* value < 0.05 was considered significant in two-tailed tests. All calculations were performed using SAS version 9.1 (SAS Institute Inc., Cary, NC).

## Results

**Fig 1:**
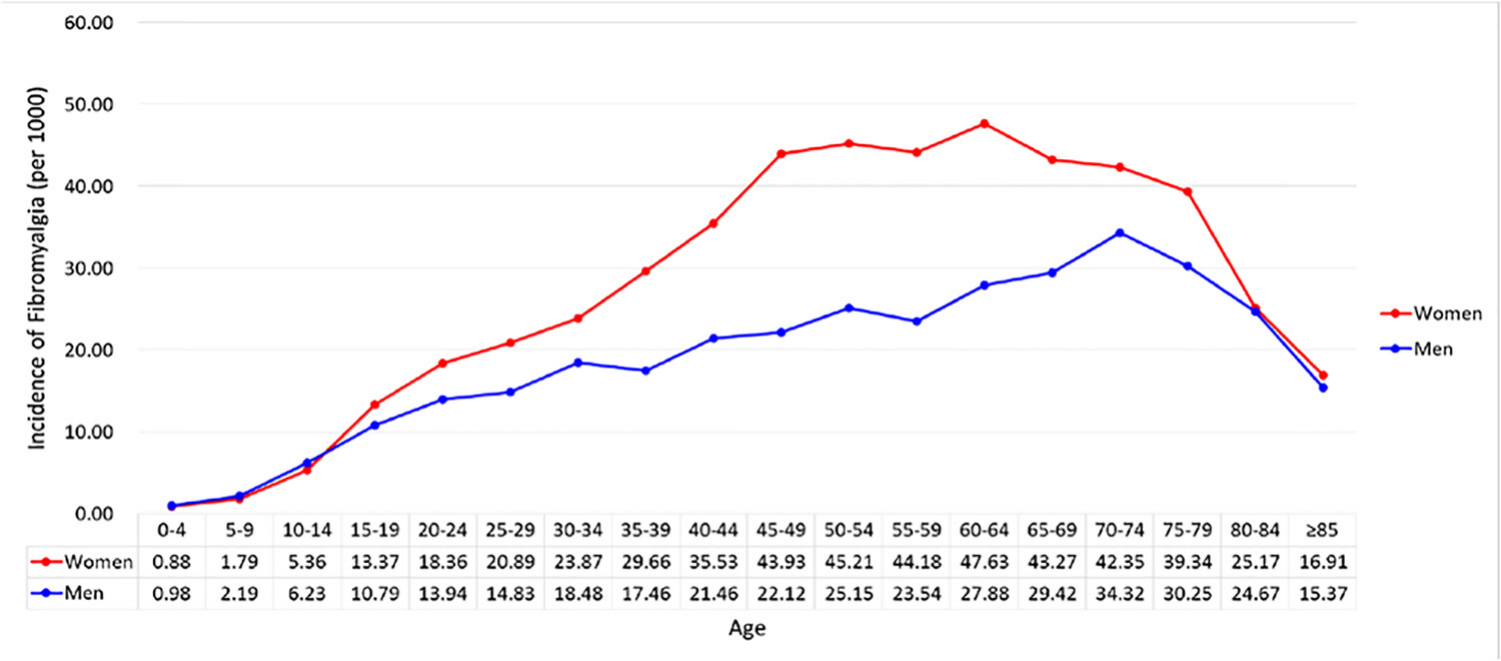
The incidence of fibromyalgia (10^3^ person-years) between women and men stratified by age subgroups.

From the LHID, we identified 61,612 fibromyalgia patients as the case cohort and 184,834 matched subjects without fibromyalgia as the reference cohort (1:3 match ratio), with a mean 8.86 ± 2.68 years (2,184,197 person-years in total) of follow-up. The standardized prevalence and incidence of fibromyalgia were 5.84 (95% CI 4.52–7.55) % and 20.2 (95% CI 13.1–31.6) per 10^3^ person-years, respectively, with respect to the Taiwanese population in 2000 [22]; the standardized fibromyalgia prevalence and incidence were 5.56 (95% CI 4.27–7.23) % and 19.2 (95% CI 12.2–30.0) per 10^3^ person-years, respectively, with respect to the World Health Organization population in 2000 [23]. Fig 1 shows the incidence of fibromyalgia in patients of both genders (per 10^3^ person-years) with respect to age (stratified by 5 years of age). The mean age of the fibromyalgia cohort was 44.1 ± 16.5 years old, with a female-to-male ratio of 6-to-4 (59.3% versus 40.7%). The highest proportion of fibromyalgia patients was in the 35–65 year-old age subgroup (57.8%). Higher proportions of fibromyalgia patients had a blue-collar status and monthly income level of TWD 15,000 to 25,000 than non-fibromyalgia subjects. The case cohort also had a significantly higher prevalence of comorbidities, including diabetes, hypertension, hyperlipidemia, congestive heart failure, cerebral vascular diseases, depression, and anxiety than the reference cohort (Table 1).

**Table 1:** Comparison of demographics and comorbidities between the fibromyalgia and non-fibromyalgia cohorts.

	Fibromyalgi		Referenc		*p*
	n = 61612		n = 184834		
	n	%	n	%	
Gender					0.99
Women	36,546	59.3	109,638	59.3	
Men	25,066	40.7	75,196	40.7	
Age, year					0.99
<35	18,456	30.0	55,368	30.0	
35–65	35,626	57.8	106,878	57.8	
>65	7,530	12.2	22,588	12.2	
Mean (SD)^#^	44.1 (16.5)		44.0 (16.5)		0.35
Occupation					< .0001
White collar	31,607	51.3	100,759	54.5	
Blue collar	24,913	40.4	67,541	36.5	
Other	5,092	8.26	16,534	8.95	
Monthly income (TWD)					< .0001
<15,000	14,029	22.8	46,873	25.4	
15,000–25,000	35,197	57.1	99,408	53.8	
>25,000	12,386	20.1	38,553	20.9	
Comorbidity					
Diabetes	5,694	9.24	12,690	6.87	< .0001
Hypertension	13,112	21.3	29,788	16.1	< .0001
Hyperlipidaemia	9,091	14.8	16,439	8.89	< .0001
Congestive heart failure	693	1.12	1,684	0.91	< .0001
Cerebral vascular diseases	4,817	7.82	9,704	5.25	< .0001
Depression	2,067	3.35	3,077	1.66	< .0001
Anxiety	4,489	7.29	5,492	2.97	< .0001

TWD, Taiwanese dollar

Chi-square test

^#^ Student’s t-test

White collar: civil services, institutional workers, enterprise, business and industrial administration personnel; Blue collar: farmers, fishermen, vendors, and industrial laborers; Other: retired, unemployed, and low-income populations

**Fig 2:**
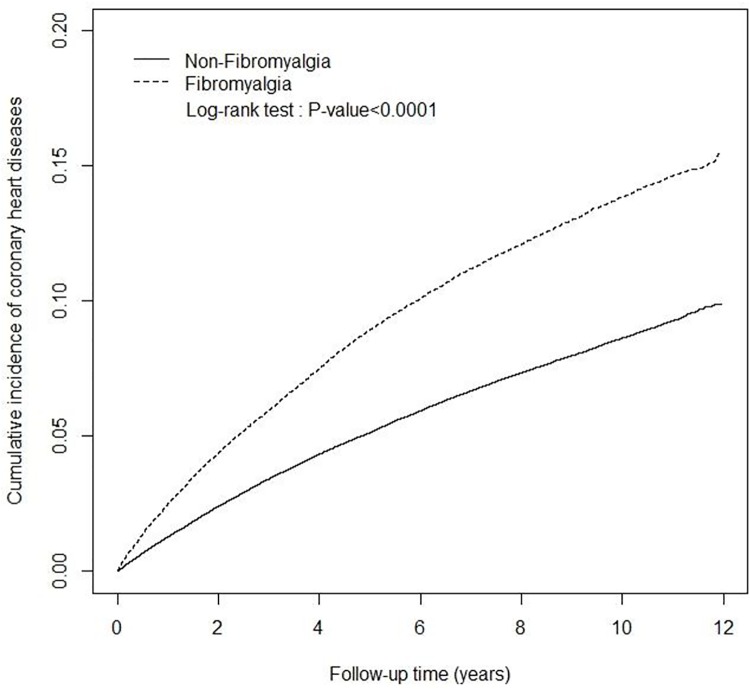
Cumulative incidence of coronary heart diseases between the fibromyalgia cohort (dashed line) and non-fibromyalgia cohort (solid line).

Fig 2 shows the cumulative incidence rates of CHD in the case and reference cohorts during 12 years of follow-up (log-rank test, *p* < 0.01). In these cohorts, 8,280 fibromyalgia patients (15.2 per 10^3^ person-years) and 15,162 reference subjects (9.26 per 10^3^ person-years) developed CHD, with fibromyalgia patients having a significant incidence rate ratio (IRR) of 1.64 (95% CI 1.61–1.68) and HR of 1.47 (95% CI 1.43–1.51) relative to the reference subjects, after adjusting for age, gender, occupation, monthly income, comorbidities of traditional cardiovascular risk factors, depression, and anxiety (Table 2).

**Table 2:** Incidence and adjusted hazard ratio for coronary heart disease stratified by gender, age, occupation, monthly income, and comorbidity status for patients with fibromyalgia and compared to those without fibromyalgia.

	Fibromyalgia			Reference				
Characteristics	Event	PY	Rate	Event	PY	Rate	IRR	Adjusted HR^†^
							(95% CI)	(95% CI)
All	8,280	544,629	15.2	15,162	1,638,194	9.26	1.64(1.61–1.68)*	1.47(1.43–1.51)*
Gender								
Female	4,796	326,677	14.7	8,641	986,356	8.76	1.68(1.63–1.72)*	1.50(1.45–1.55)*
Male	3,484	217,952	16.0	6,521	651,838	10.0	1.60(1.54–1.65)*	1.44(1.38–1.50)*
Age, years								
< 35	342	177,075	1.93	355	517,114	0.69	2.81(2.68–2.95)*	2.40(2.06–2.79)*
35–64	5,360	317,450	16.9	9,501	969,430	9.80	1.72(1.68–1.77)*	1.45(1.40–1.50)*
> 65	2,578	50,104	51.5	5,306	151,650	35.0	1.47(1.39–1.55)*	1.36(1.30–1.43)*
Occupation								
White collar	3,496	285,382	12.25	6,904	908,254	7.60	1.61(1.56–1.66)*	1.48(1.42–1.55)*
Blue collar	3,948	216,622	18.23	6,781	590,683	11.48	1.59(1.53–1.64)*	1.45(1.39–1.51)*
Other	836	42,625	19.61	1,477	139,256	10.61	1.85(1.72–1.99)*	1.54(1.41–1.68)*
Monthly income, TWD								
< 15,000	1,915	120,088	15.95	3,880	400,599	9.69	1.65(1.57–1.72)*	1.49(1.41–1.57)*
15,000–-25,000	4,832	311,949	15.49	8,339	882,458	9.45	1.64(1.59–1.69)*	1.48(1.43–1.53)*
>25,000	1,533	112,592	13.62	2,943	355,137	8.29	1.64(1.57–1.72)*	1.43(1.35–1.53)*
Comorbidity								
No	2,323	365,924	6.35	5,399	1,280,423	4.22	1.51(1.46–1.55)*	1.68(1.60–1.76)*
Yes	5,957	178,704	33.3	9,763	357,770	27.3	1.22(1.18–1.27)*	1.34(1.30–1.39)*

PY, person-years; Rate, incidence rate (per 1,000 person-years); IRR, incidence rate ratio; HR, hazard ratio

^†^ Adjusted HR: multiple analysis after adjusting for age, gender, occupation, monthly income, and comorbidities

*p<0.001

Although women were more likely to have fibromyalgia early in life compared with men (Fig 1), both genders of fibromyalgia patients had comparable risks for developing CHD. The incidence rates increased with age in both cohorts (Table 2); however, it is noteworthy that the highest age-specific CHD risk in fibromyalgia patients was in the youngest subgroup (age < 35 years: adjusted HR [aHR] 2.40, 95% CI 2.06–2.79), with steadily declining estimates as age increased. Finally, our analysis showed that CHD risks in fibromyalgia patients relative to non-fibromyalgia reference subjects were comparable across different occupation statuses and monthly income levels.

The impact of comorbidities on outcomes for fibromyalgia patients was assessed by stratifying the case and reference cohorts with respect to comorbidity status. CHD risks were significant in both fibromyalgia patients with (aHR 1.34, 95% CI 1.30–1.39) and without (aHR 1.68, 95% CI 1.60–1.76) comorbidities relative to their respective matched reference subjects (Table 2). We further stratified fibromyalgia patients and reference subjects with respect to comorbidity status (Table 3). In fibromyalgia patients without and with comorbidities, the aHRs for CHD ranged from 1.60–1.76 and 1.30–1.39, respectively; the HRs were significantly greater than those of their respective reference subjects.

**Table 3:** Incidence and adjusted hazard ratio for coronary heart diseases stratified by comorbidity for the fibromyalgia cohort and compared with the non-fibromyalgia cohort.

	Fibromyalgia			Reference				
Characteristics	Event	PY	Rate	Event	PY	Rate	IRR (95% CI)	Adjusted HR^†^(95% CI)
Diabetes								
No	6,457	502,818	12.8	11,945	1,545,637	7.73	1.66(1.62–1.70)**	1.51(1.46–1.56)**
Yes	1,823	41,810	43.6	3,217	92,556	34.8	1.25(1.17–1.34)**	1.29(1.22–1.37)**
Hypertension								
No	3,898	449,503	8.67	7,561	1,419,868	5.33	1.63(1.59–1.67)**	1.59(1.53–1.66)**
Yes	4,382	95,125	46.1	7,601	218,326	34.8	1.32(1.27–1.38)**	1.34(1.29–1.39)**
Hyperlipidemia								
No	5,587	473,821	11.8	11,315	1,509,897	7.49	1.57(1.54–1.61)**	1.50(1.45–1.55)**
Yes	2,693	70,808	38.0	3,847	128,297	30.0	1.27(1.20–1.34)**	1.35(1.29–1.42)**
Congestive heart failure								
No	7,982	540,580	14.8	14,615	1,629,147	8.97	1.65(1.61–1.68)**	1.48(1.44–1.52)**
Yes	298	4,049	73.6	547	9,047	60.5	1.22(1.02–1.46)*	1.25(1.08–1.44)*
Cerebral vascular diseases								
No	6,812	509,627	13.4	12,994	1,572,683	8.26	1.62(1.58–1.66)**	1.47(1.43–1.52)**
Yes	1,468	35,002	41.9	2,168	65,511	33.1	1.27(1.17–1.37)**	1.40(1.31–1.50)**
Depression								
No	7,872	527,718	14.9	14,754	1,613,888	9.14	1.63(1.60–1.67)**	1.46(1.42–1.50)**
Yes	408	16,911	24.1	408	24,305	16.8	1.44(1.25–1.65)**	1.67(1.45–1.90)**
Anxiety								
No	7,294	508,421	14.35	14,162	1,595,256	8.88	1.62(1.58–1.65)**	1.47(1.43–1.51)**
Yes	986	36,208	27.23	882	44,312	19.9	1.37(1.24–1.51)**	1.57(1.43–1.72)**

PY, person-year; Rate, incidence rate (per 1,000 person-years); IRR, incidence rate ratio; HR, hazard ratio

^†^Adjusted HR: multiple analysis after adjusting for age, gender, occupation, income and comorbidities

*p<0.05

**p<0.001

**Fig 3:**
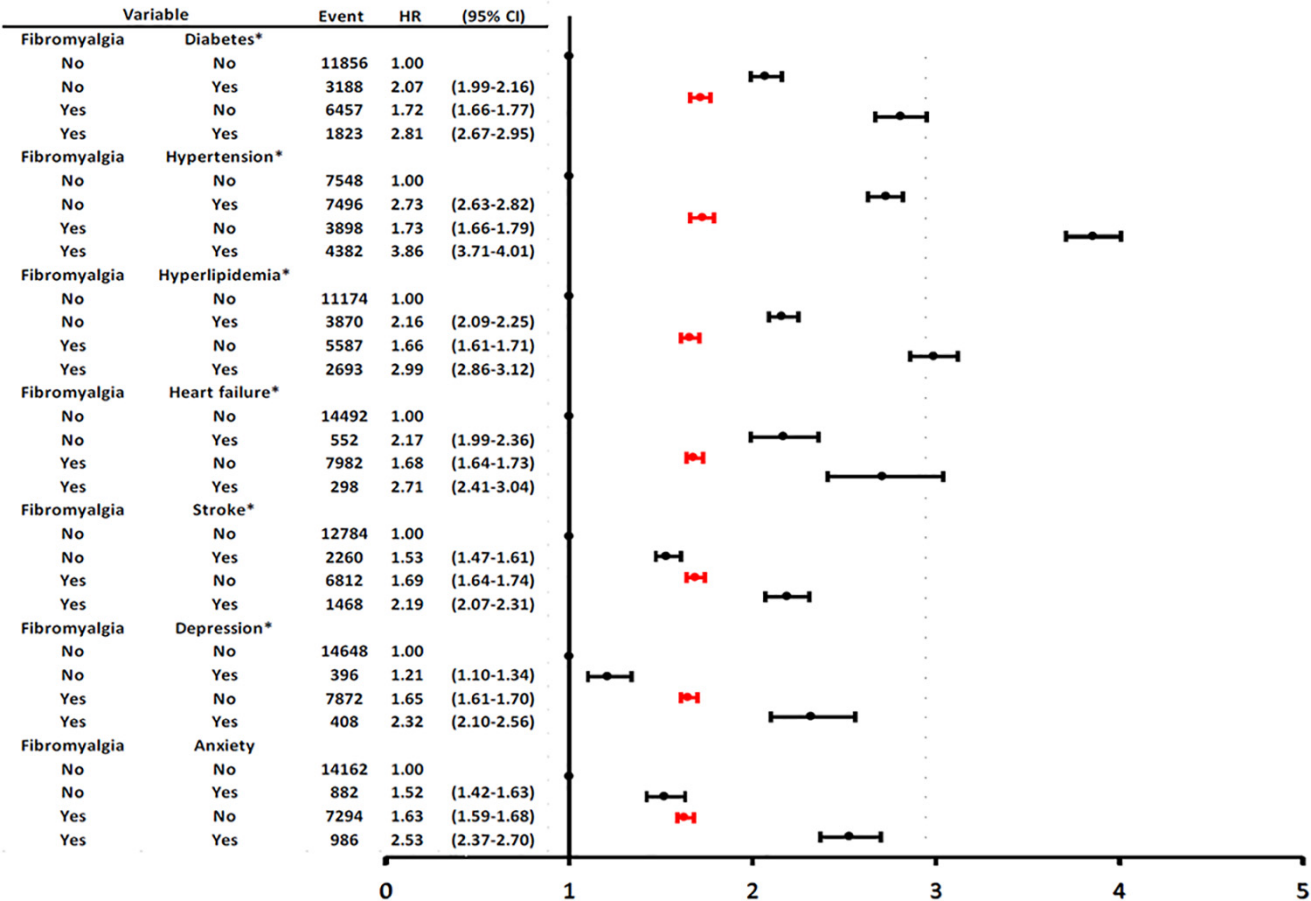
Adjusted hazard ratios for coronary heart diseases associated with the fibromyalgia-comorbidity interaction. The first line indicates patients with the respective comorbidity. The second and red line indicates patients with primary fibromyalgia, and the third line indicates fibromyalgia patients with the respective concomitant comorbidity. *p for interaction < 0.01.

The joint effect on CHD risk and interaction between fibromyalgia and the respective comorbidity are shown in Fig 3. Relative to reference subjects without fibromyalgia and comorbidities, fibromyalgia patients without respective comorbidity of diabetes, hypertension, hyperlipidemia or congestive heart failure had aHRs for CHD ranging from 1.66–1.72, which were generally lower than the respective risk ranging from 2.16–2.75 in non-fibromyalgia patients but with these traditional cardiovascular comorbidities (*p* for interaction < 0.01, respectively). It is noteworthy that relative to reference subjects of no depression, no fibromyalgia, the CHD risk in patients with fibromyalgia but without depression (aHR 1.65, 95% CI 1.61–1.70) was higher than that in patients with depression but without fibromyalgia (aHR 1.21, 95% CI 1.10–1.34). However, the CHD risks between patients with fibromyalgia but without anxiety and patients with anxiety but without fibromyalgia were comparable. The effect of combing fibromyalgia with comorbidity of either diabetes, hypertension, hyperlipidemia, congestive heart failure, cerebral vascular diseases, depression or anxiety was associated with significantly higher CHD risk than that in patients with fibromyalgia only and that in those with respective comorbidity.

## Discussion

The present study confirmed that fibromyalgia patients had a 47% increased risk of CHD after adjusting for age, gender, occupation, monthly income, and comorbidities. Although the impact of fibromyalgia on developing CHD was moderate, the risk was not as high as that for traditional cardiovascular risk factors of diabetes, hypertension, hyperlipidemia or congestive heart failure. A greater CHD risk was noted in fibromyalgia patients with concomitant comorbidities than in patients with primary fibromyalgia or in reference patients with the respective comorbidity alone.

Several limitations were noted in this study. First, fibromyalgia patients may have been misclassified in the LHID population, because patients in the case cohort with a clinical diagnosis of fibromyalgia defined by the physicians did not necessarily meet the classification criteria of American College of Rheumatology [3, 4]. However, this current study demonstrated a higher standardized prevalence rate and a greater male-to-female ratio in Taiwanese fibromyalgia patients, which findings were similar to the reports in the United States (6.4%) [13], Germany (5.4%) [12] and Nantou County, Taiwan (6.7%) [24] using the modified 2010 classification criteria of American College of Rheumatology to survey for the general population [11]. The new criteria integrates the widespread pain index and self-reported specific symptoms may facilitate epidemiological investigations and should help to identify more male and younger patients [25].

Second, the criteria to diagnose fibromyalgia include depression. A population-based study in the United Kingdom reported 16.7% of patients with chronic widespread pain (classified using ACR criteria for fibromyalgia) had mental disorders [26]. A risk of CHD was recently reported in patients with depressive disorder [27], and CHD has been associated with subsequent depression [28]. In this study, we showed an increased risk of CHD in individuals with depression, which is compatible with previous report [27]. We also found the adjusted HR for CHD in patients with primary fibromyalgia (aHR 1.65) was significantly higher than that in depressed patients without fibromyalgia (aHR 1.21) and the risk of CHD between patients with fibromyalgia and those with anxiety was comparable (aHR 1.63 versus 1.52). However, the differentiating among anxiety, depressive mood and major depression disorder in fibromyalgia patients can be vague, and we need to interpret the results with caution.

Third, the LHID does not provide parameters, including pain scores, laboratory data on inflammatory index, metabolic profiles, body mass index, and personal habits such as cigarette smoking and alcohol drinking. Therefore, we could not correlate body mass index and degree of pain [29] or assess the impact of smoking [30], which are known risk factors for fibromyalgia or CHD. However, Taiwanese women have a very low smoking rate (4.3%) compared with that in men (46.8%) [31]; it is worth noting that females and young children can be exposed to high environmental tobacco exposure due to second hand smoking [32]. Nonetheless, the impact of second hand smoking on the development of fibromyalgia, as we considered, should be far less than that on the development of CHD. Even though there is a risk of CHD due to second hand smoking, the evidenced risk of fibromyalgia resulting from second hand smoking so far is limited. Therefore, we assumed the bias that fibromyalgia patients might have CHD due to second hand smoking can be non-differential and this bias is not likely to confound fibromyalgia risk in female patients.

Fourth, patients with fibromyalgia may show an increased health-care-seeking behavior, which can explain our results that patients with fibromyalgia had significantly higher prevalence of comorbidities than the reference cohort, and may have impacted the CHD risk. Nevertheless, the independent risk of fibromyalgia for CHD was confirmed by sequential stratifications by comorbidity in both patients and reference subjects, and CHD risk persisted in fibromyalgia patients without comorbidities and was higher in younger patients.

Fifth, one could argue that CHD risk in fibromyalgia patients can be potentiated by chronic use of anti-depressants and painkillers, including non-steroidal anti-inflammatory drugs (NSAIDs) and cyclooxygenase-2 inhibitors (coxibs), which have been suggested to enhance cardiovascular risk. However, a population-based study showed that the rate of cardiovascular events was significantly higher for patients who took rofecoxib (RR 1.15) and was also significantly lower for those who took naproxen (RR 0.75) compared with the reference patients who did not use NSAIDs or coxibs [33]. Moreover, as indicated in a population-based cohort study [27] that CHD risk of depressed patients was not increased by antidepressants, which are commonly used in fibromyalgia treatment. Although we were unable to put all these treatment into modeling, these reports may suggest that painkillers and antidepressants did not lead to important modifications in patients’ cardiovascular risk [33]. People can still argue that other treatment modality or residual confounding factors can exit. For instance, although acupuncture has been used to treat pain and fibromyalgia [34–36], it has no reported risk to CHD. Whether traditional Chinese herbs can be used to deal with fibromyalgia and whether they might increase the risk of CHD are undetermined [37]. In light of the huge national insurance data and large sample size, these confounders may become non-differential bias and the evidences demonstrated above may strengthen the contention that fibromyalgia can be an independent risk factor for CHD.

Several lines of evidence can provide a possible biological explanation to support the increased CHD risk in fibromyalgia patients. First, people with chronic widespread pain have been shown in population-based studies to have greater psychological distress and mental disorders than those without pain [26, 38, 39]. Previous reports indicated that people with chronic diseases, such as fibromyalgia and CHD, have a common life experience of childhood abuse, from a perspective of mind-body medicine [40]. The potential role of early psychosocial adversities in the vulnerability of fibromyalgia and CHD should be considered and appropriate referral may be needed for follow-up counseling [41]. Second, fibromyalgia was previously shown to be significantly associated with degree of stress [24], and chronic stress contributes to persistent activation of the sympathetic nervous system and hypothalamic-pituitary-adrenal axis [42]. The autonomic dysfunction in patients with fibromyalgia is characterized by stronger parasympathetic decline [43] and maintains patients in a state of sympathetic hyperactivity [44]. Indeed, evidence showed that fibromyalgia patients with exaggerated sympathetic modulation of the sinus node and enhanced heart rate variability [45, 46] may incur the risk of CHD.

The strengths of this study warrant mention. First, the study data source, Taiwan’s NHIRD enrolls over 22 million citizens and employees in a national insurance program. The stringent NHI surveillance program, which rigorously monitors and audits insurance reimbursement claims to prevent healthcare fraud, strengthens the reliability of diagnoses based on insurance claims. Second, the characteristics of our fibromyalgia patients were consistent with previous reports of female predominance and the majority being middle-aged [5–8, 10, 24]. The similarity of these characteristics supports the validity of identifying fibromyalgia patients from the LHID and of generalizing and applying our results on a representative sample of Taiwan’s general population to other populations. Third, a recent Taiwanese cohort study reported a two-fold increased risk of CHD in fibromyalgia patients relative to the reference subjects [47]. However, the reported risk of chronic pain was unexpectedly exceeding that of traditional risk factors. Our study using the large sample for subgroup analysis ascertained the impact of fibromyalgia on CHD. Moreover, a markedly enhanced risk of CHD was evidenced by the synergistic effect of fibromyalgia with each individual comorbidity, in correspondence with previous report (47). Fourth, the adequate study power and the long patient observation period raised the potential for fibromyalgia to predispose patients to develop CHD.

In conclusion, our study finds that fibromyalgia is an independent risk for CHD development. However, the risk of primary fibromyalgia is not as high as the traditional cardiovascular risk, but its impact on CHD is significantly higher than that of depression. A noteworthy finding is the markedly enhanced risk of CHD in fibromyalgia patients with concomitant comorbidities.

## Supporting Information

S1 Text Data Availability Statement. (DOCX)

S2 Text Ethics Statement. (DOCX)
