# Supplementary material for: Increased Risk of Coronary Heart Disease in Patients with Primary Fibromyalgia and Those with Concomitant Comorbidity—A Taiwanese Population-Based Cohort Study
Source: PLoS One. 2015 Sep 14;10(9):e0137137. doi: 10.1371/journal.pone.0137137 (PMC4569466; doi:10.1371/journal.pone.0137137)
Supplement: S1 Text — (DOCX) [file pone.0137137.s001.docx]

**S1 Text. Data Availability Statement**

All data and related metadata were deposited in an appropriate public repository. The data on the study population that were obtained from the NHIRD (<http://w3.nhri.org.tw/nhird//date_01.html>) are maintained in the NHIRD (<http://nhird.nhri.org.tw/>). The NHRI is a nonprofit foundation established by the government. These data were released by the NHIRD for research uses. Every interested researcher is able to obtain the data in the same way that we did.
