# Supplementary material for: Increased Risk of Coronary Heart Disease in Patients with Primary Fibromyalgia and Those with Concomitant Comorbidity—A Taiwanese Population-Based Cohort Study
Source: PLoS One. 2015 Sep 14;10(9):e0137137. doi: 10.1371/journal.pone.0137137 (PMC4569466; doi:10.1371/journal.pone.0137137)
Supplement: S2 Text — (DOCX) [file pone.0137137.s002.docx]

**S2 Text. Ethics Statement**

The NHIRD encrypts patient personal information to protect privacy and provides researchers with anonymous identification numbers associated with relevant claims information, including sex, date of birth, medical services received, and prescriptions. We excluded all individually identifying or patient demographic information.  Therefore, the patient consent is not required to access the NHIRD. This study was approved by the Institutional Review Board (IRB) of China Medical University (CMU-REC-101-012). The IRB specifically waived the consent requirement.
